# Automatic Root Length Estimation from Images Acquired In Situ without Segmentation

**DOI:** 10.34133/plantphenomics.0132

**Published:** 2024-01-12

**Authors:** Faina Khoroshevsky, Kaining Zhou, Sharon Chemweno, Yael Edan, Aharon Bar-Hillel, Ofer Hadar, Boris Rewald, Pavel Baykalov, Jhonathan E. Ephrath, Naftali Lazarovitch

**Affiliations:** ^1^ Department of Industrial Engineering and Management, Ben-Gurion University of the Negev, Beer Sheva, Israel.; ^2^ The Jacob Blaustein Center for Scientific Cooperation, The Jacob Blaustein Institutes for Desert Research, Ben-Gurion University of the Negev, Sede Boqer, Israel.; ^3^ The Albert Katz International School for Desert Studies, The Jacob Blaustein Institutes for Desert Research, Ben-Gurion University of the Negev, Sede Boqer, Israel.; ^4^ Department of Communication Systems Engineering, School of Electrical and Computer Engineering, Ben-Gurion University of the Negev, Beer Sheva, Israel.; ^5^ Institute of Forest Ecology, Department of Forest and Soil Sciences, University of Natural Resources and Life Sciences, Vienna (BOKU), Vienna, Austria.; ^6^ Faculty of Forestry and Wood Technology, Mendel University in Brno, Brno, Czech Republic.; ^7^ Vienna Scientific Instruments GmbH, Alland, Austria.; ^8^ French Associates Institute for Agriculture and Biotechnology of Drylands, The Jacob Blaustein Institutes for Desert Research, Ben-Gurion University of the Negev, Sede Boqer, Israel.

## Abstract

Image-based root phenotyping technologies, including the minirhizotron (MR), have expanded our understanding of the in situ root responses to changing environmental conditions. The conventional manual methods used to analyze MR images are time-consuming, limiting their implementation. This study presents an adaptation of our previously developed convolutional neural network-based models to estimate the total (cumulative) root length (TRL) per MR image without requiring segmentation. Training data were derived from manual annotations in Rootfly, commonly used software for MR image analysis. We compared TRL estimation with 2 models, a regression-based model and a detection-based model that detects the annotated points along the roots. Notably, the detection-based model can assist in examining human annotations by providing a visual inspection of roots in MR images. The models were trained and tested with 4,015 images acquired using 2 MR system types (manual and automated) and from 4 crop species (corn, pepper, melon, and tomato) grown under various abiotic stresses. These datasets are made publicly available as part of this publication. The coefficients of determination (*R*^2^), between the measurements made using Rootfly and the suggested TRL estimation models were 0.929 to 0.986 for the main datasets, demonstrating that this tool is accurate and robust. Additional analyses were conducted to examine the effects of (a) the data acquisition system and thus the image quality on the models’ performance, (b) automated differentiation between images with and without roots, and (c) the use of the transfer learning technique. These approaches can support precision agriculture by providing real-time root growth information.

## Infographic

**Figure:**
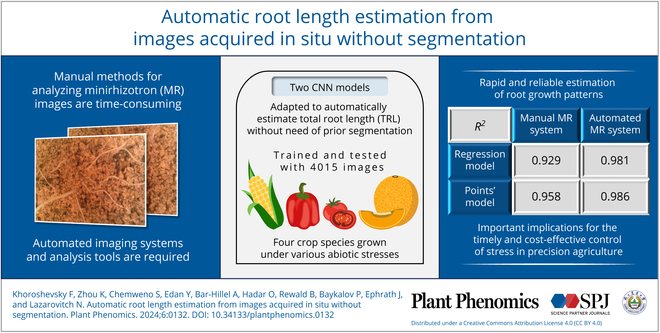

## Introduction

Prevailing stresses caused by climate change, such as drought and salinity, put substantial constraints on crop yields and thus pose threats to food security and economic development [1]. Root traits (e.g., root length and rooting depth) related to nutrient and water acquisition play critical roles in stress tolerance, given their high levels of plasticity [2–4]. Therefore, trait-based root phenotyping has been proposed as a promising approach for crop selection and improvement [5–9]. The spatial distribution of the roots and their growth is very sensitive to various physical, chemical, and biological factors, as well as to the hydraulic properties of the soil that affect the availability of water, nutrients, and oxygen for plants [10–12]. Therefore, it is important to describe root growth under the influence of diverse environmental conditions to accurately understand agricultural systems and to develop modeling capabilities for decision-making.

Investigation of root dynamics in response to changing environments has been achieved with image-based root phenotyping techniques [13,14]. Among these, the minirhizotron (MR) technique has been widely used for the nondestructive in situ observation of roots [15]. The MR consists of a transparent observation tube embedded in the root zone and an image acquisition component that allows images to be collected repeatedly, allowing the fates of individual roots to be followed through time [15]. Information about roots (e.g., length, diameter, and mortality) can be extracted from MR images using image analysis programs such as Rootfly (Wells and Birchfield, Clemson University, South Carolina, USA), RootPainter [16], and WinRHIZO (Régent Instruments, Quebec, Canada). Root length is especially important since it allows us to calculate various temporal and spatial root parameters based on it, such as rooting depth, root length density (RLD), and root growth rate. A major drawback of the MR technique is that the conventional image collection and analysis processes are performed manually and are therefore time-consuming, which considerably limits the size and number of experiments that can be reasonably conducted [17]. Moreover, the outcomes of image analyses are subjective and thus dependent on the knowledge and experience of the annotator [18,19]. Automated imaging systems and analysis tools are required to overcome these challenges.

In recent years, deep learning (DL) algorithms [20], a subset of machine learning tools, have emerged as a driving force to provide state-of-the-art performance in image-based plant phenotyping [21,22], especially in plant stress phenotyping [23,24]. DL tools are increasingly used by plant scientists to process large datasets of images collected using high-throughput phenotyping platforms, facilitating recent developments in the automation of the agricultural domain [21,25,26]. More specifically, convolutional neural network (CNN)-based architectures have become very popular because of their excellent performance in complex computer vision tasks [27–30]. The ability of CNNs to learn features directly from data without prior knowledge has allowed them to overcome the limitations of traditional machine learning approaches.

Existing CNN tools have been used for analysis of root images taken from artificial plant cultivation systems such as glass tubes filled with transparent gellan gum [31] or growth pouches in a controlled environment chamber with individual pouches transferred to a copy stand for imaging [32]. Analyzing root systems on images taken in situ is more difficult for 3 reasons: (a) the low contrast between the roots and the heterogeneous rhizosphere; (b) the inconsistent scene illumination caused by the light source, soil water content, and soil type; and (c) the various artifacts, such as scratches or water bubbles on the wall of observation tube. These issues have limited the automated analysis of roots in MR images using the available CNN models [18].

Current CNNs used for root image analysis, predominantly rely on segmentation-based architectures [33–36]. Segmentation requires that each input image is paired with a set of labels corresponding to each of the pixels in the input image [16,35]. These models require considerable human effort to annotate the segmentation masks for model training, i.e., to mark the contour of each root in the annotation tool to obtain all the relevant pixels of the roots in the image. Creating such dense per-pixel annotations for training is a time-consuming process [16].

In this paper, we propose 2 fully automated CNN-based MR image analysis models to estimate the total root length (TRL) in each image, without requiring root segmentation annotation for training. TRL was calculated as the sum of root length per image. We compare our models to 2 recent studies [16,19] that also perform automated root length estimation, which rely on models that learn to perform root segmentation as a preliminary step prior to root length estimation. Smith et al. [16] presented RootPainter, an open-source graphical-user-interface-based software for creating a dataset and training a fully convolutional network, a modified version of U-Net [37], to perform image segmentation. They use this tool to train a model to perform root segmentations of rhizotron-based images and to obtain root length estimates by doing skeletonization of generated segmentations and pixel counting. Bauer et al. [19] incorporated a pipeline for TRL estimation by combining open-source software tools that included root segmentation with the RootPainter [16], followed by extracting TRL estimates from the resulting segments. This was achieved with RhizoVision Explorer [38] by the sum of the Euclidean distances between the connected skeletal pixels of the root topology.

**Fig. 1.**
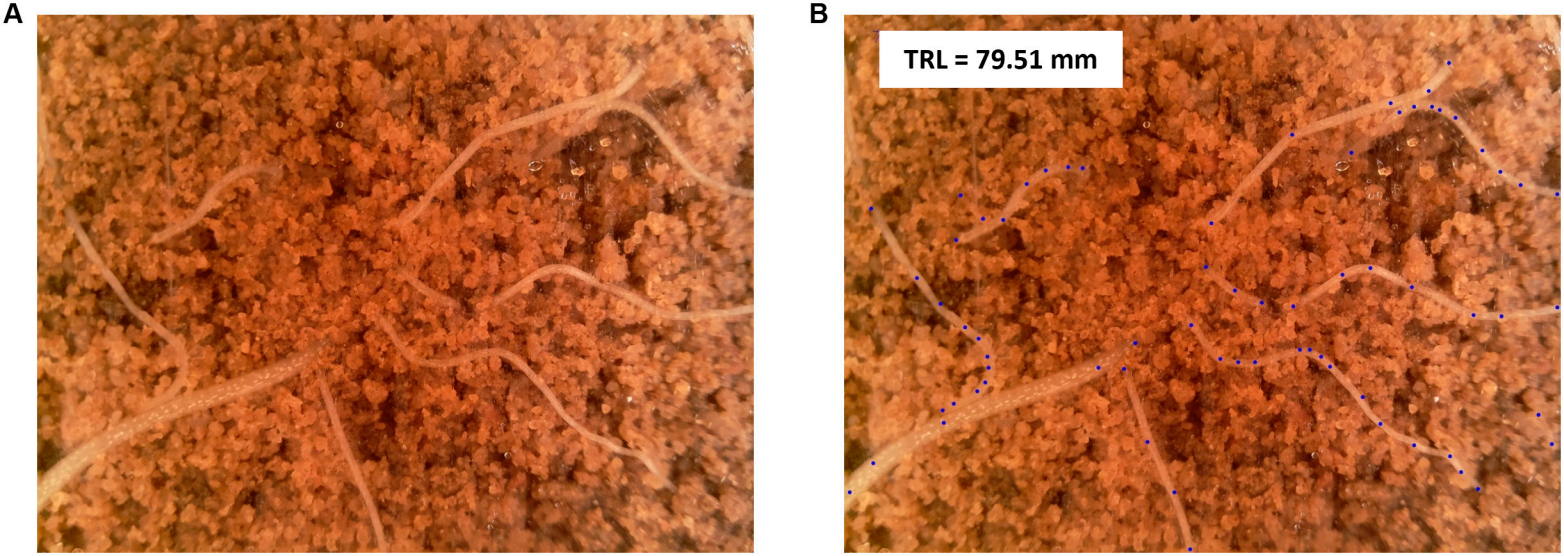
Example of (A) a raw root image and (B) the same image with added GT point annotations drawn on top and the resulting TRL value from Rootfly.

The “ground-truth” (GT) values for training the 2 models we propose were acquired using Rootfly, a commonly used manual MR image analysis software (Fig. S1). Rootfly allows us to estimate the TRL values based on the points coordinates that the annotator marks along the entire length of each root during the annotation process (Fig. 1).

Both the TRL values and the coordinates of these points in each MR image are referred to as GT values in this study. Our suggested models [39] for automated TRL estimation only require the inherited information provided by Rootfly for training, without additional annotations from other software.

The first suggested model is a regression-based network, which implements a function that maps the entire image to a single value that represents a specific attribute of the image (here, the TRL value). Only the GT value of TRL per image is required to train this model. The second model is based on explicit object detection that requires the GT values of both the TRL and the coordinates of the points per image for training. In general, a detection task can be based on the detection of a bounding box around each object in the image [40,41] or on the detection of an object’s central point [42]; however, in the suggested model, it refers to the detection of the points along the roots. The detection of these points also allows visual inspection of the roots in the image—once there is a trained model that automatically outputs its estimates of the coordinates of the root points, the annotator can use them to recheck their annotations. Since human annotations are inconsistent and subjective [16,43], the proposed visual inspection feature could reduce errors in annotations leading to improved annotation quality. In addition, the performance of current semiautomated and automated software used for root image analysis has been measured on small datasets [16,31–33] and on a limited range of species each time (e.g., corn, wheat, and rice). These limitations have led to overfitting issues when new root images are introduced [44]. An important method for handling this kind of difficulty is transfer learning, in which a base network is trained using a large dataset and then used for another task that has a smaller dataset [45]. A trained network can be used for feature extraction on the basis of one dataset and then used for another dataset, e.g., when the features of a CNN that were trained using a simulated x-ray image of a soil–root system are used for another model applied to real images [46]. Alternatively, transfer learning can be done using the first trained layers of the base (trained) network as part of a new target network, while the rest of the layers are randomly initialized and trained toward the target task [45]. The copied layers can then be fine-tuned or left “frozen” (i.e., not altered during training on the new task). We examine the contribution of the transfer learning technique using the later approach with an entire trained CNN being fine-tuned for a new dataset. We also contribute by making publicly available the datasets that were acquired in this study. This includes 4,015 images with the corresponding TRLs and points’ coordinate annotations [47].

The objectives of this study were to (a) adapt 2 existing CNN models to the TRL estimation task (a completely different task without segmentation annotation); (b) examine the influence of sample size and image quality (which depend on the image acquisition system) on the models’ performance; (c) suggest a method for distinguishing between images with and without roots; (d) demonstrate the models’ contributions to root research by comparing RLD plots generated on the basis of the models’ TRL estimates and the GT annotations from Rootfly; (e) examine the transfer learning opportunities when testing a trained model on a new dataset with different properties; and (f) make publicly available a new MR dataset acquired by 2 types of MR systems (manual and automated) with high-quality annotations.

The rest of the paper is organized as follows: the “Materials and Methods” section describes the models’ architectures, data acquisition, datasets, and evaluation, followed by the “Results” section. The “Discussion” section provides guidelines based on exploration of the results. The paper is summarized in the “Conclusion” section.

## Materials and Methods

### Architectures of the models

**Fig. 2.**
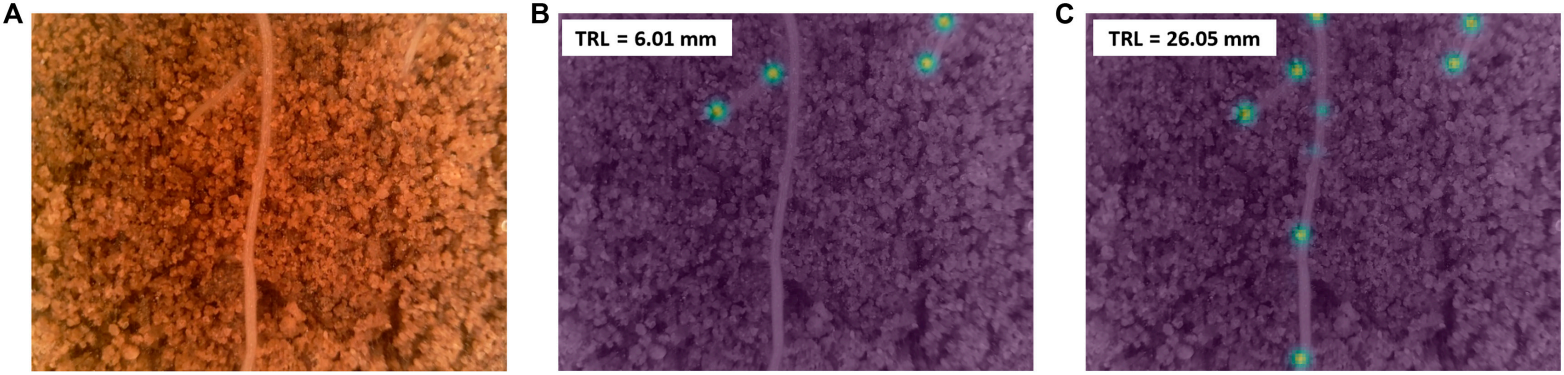
Comparison of a GT heatmap of a root’s points (human annotations) and the heatmap generated by the points model. (A) Raw MR image. (B) The GT heatmap of the points coordinates on top of the raw image and its corresponding TRL value. The heatmap was generated on the basis of the points manually annotated in Rootfly. (C) The predicted heatmap on top of the raw image and the corresponding TRL value.

The 2 suggested CNN models (Table S1 and Fig. S2) were based on CNN models we developed for the task of part counting [39] that were implemented to count the number of leaves on a single plant in an image [39,48]. In later studies, they were used as alternative options in a 2-stage network for counting parts per object in an image with multiple objects [49,50], in which the objects were first detected using the RetinaNet architecture [40], and for each detection, these models were used for part counting of the detected object. In the present study, these 2 models were adapted for the completely different task of TRL estimation, with multiple objects (roots) in an image. The first model is a multiple-scale regression model, which is based on direct regression and is designated the regression model. The second model is a detection and regression (D+R) model, designated the points model, which learns to detect the coordinates of points along the roots in the image and uses them to output the TRL estimates. Although the architecture of the models remains the same [39], the training data are different in concept. The regression model outputs the total length of roots per image without using additional annotations for training, but, instead of receiving annotations of part count per image, it now learns to output the TRL values per image when there are multiple roots or no roots at all. The points model is trained to detect a different set of points—not the center point of parts (leaves) of a single object (a plant) in an image but points along each object (a root) when it may have multiple objects in an MR image. In addition, instead of parts counting, the model estimates the TRL value based on the detected coordinates of points along the roots (Table S1 and Fig. S2). As part of the preparation of the GT annotations for the points model, a 2-dimensional “density estimation” heatmap is generated for each image on the basis of the GT coordinates of the annotated points. This is done with a Gaussian kernel placed around the coordinates of each point, to obtain a heatmap with Gaussian distribution around each point, from which the model learns to output the TRL estimates. These maps are used in training when the heatmap generated by the model (i.e., detecting the roots) is compared with the GT heatmap and the relevant loss is calculated [39]. We generate a density estimation map instead of generating a binary map of the points’ coordinates, for 2 reasons. The first is that the specific coordinates of the points depend on the subjective annotations of the user. Given the nature of the task, the exact coordinates can vary and cannot be defined with pixel-level precision. Second, it is more difficult to train a network to output a binary map, where even a deviation of a single pixel in the root’s estimated location implies a full error. When small deviations are considered to be partial successes, gradual learning is promoted. A trained model can generate this map by itself in test time, which can then be used for the final estimation of TRL. Figure 2B shows an example of a GT map that was generated according to the points’ annotation data derived from Rootfly on a raw MR image (Fig. 2A). The predicted map, which was generated in test time by a trained model for this image, is shown in Fig. 2C. In this example, the middle root in the image was not annotated because of an error of the annotator (Fig. 2B) but was detected by the model (Fig. 2C).

### Data acquisition

**Fig. 3.**
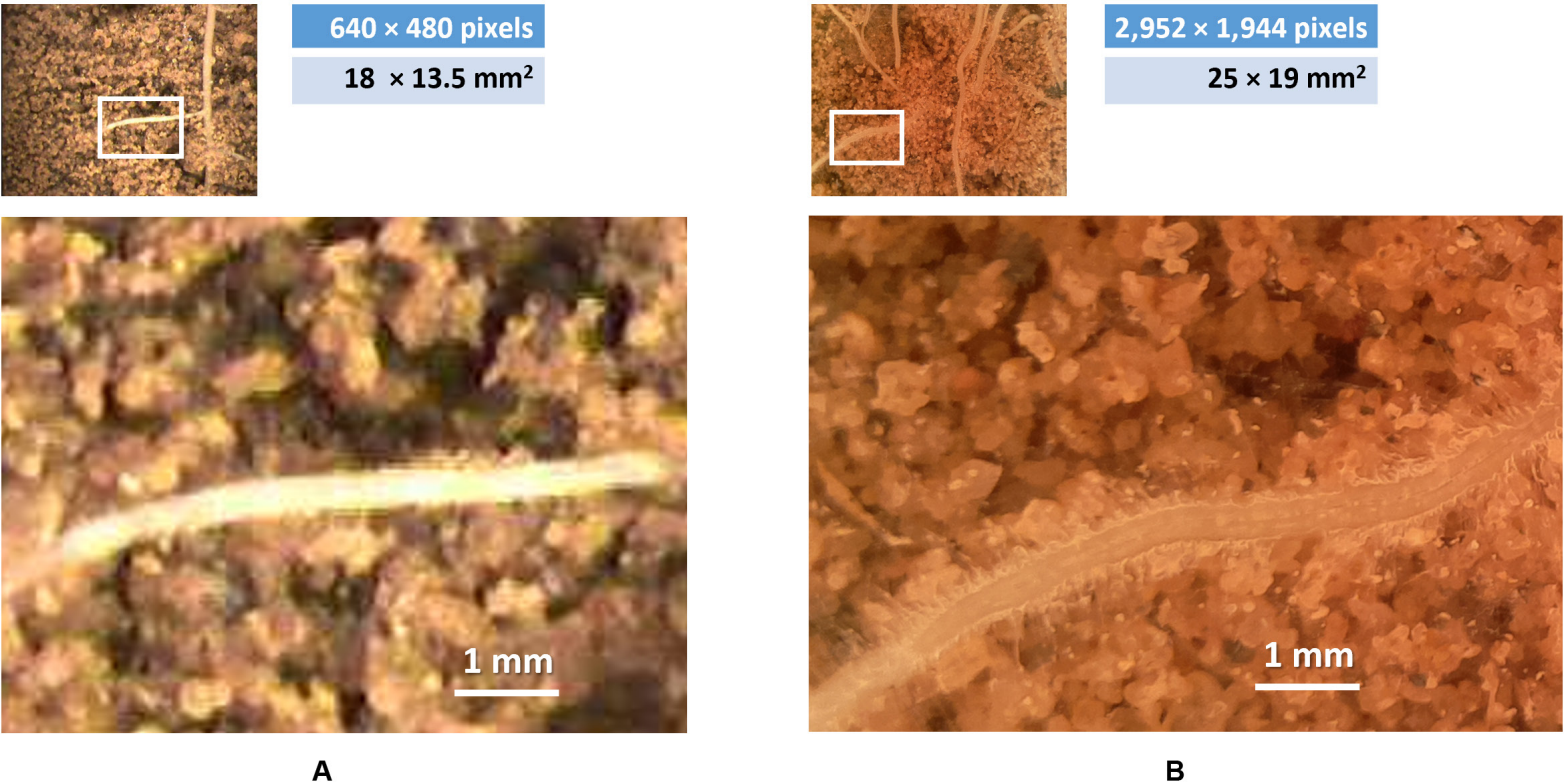
Comparison of root images taken using (A) a conventional manual MR system or (B) an automated MR system. Images in the second row are enlarged versions of the marked parts (with a white border) in the original root images in the first row.

Data were acquired with 2 MR systems, manual and automated (Fig. 3), which collectively record images that display the distribution of the root length across a range of depths in the soil profile. The automated MR system has cameras with higher image resolution, larger observation areas, and better illumination than the manual system resulting in meaningful differences in image quality.

#### Manual image acquisition

Manual image acquisition was performed using a Bartz Technology Co. (Carpinteria, California, USA) MR camera system with its default settings. The camera was attached to an indexing handle and controlled using a laptop-computer-based image capture system (I-CAP, Bartz Technology Co., Carpentaria, California, USA). Lighting was supplied by 4 small incandescent light bulbs surrounding the camera. The camera was manually lowered into an observation tube installed 10 to 15 cm in front of the measured plant. One operator moved the camera from the bottom of the tube upward, in increments of 13.5 mm, over the entire length of the tube, while another operator monitored the quality of the image on the laptop and captured it simultaneously. After all images were acquired from the measured tube, the camera and laptop were moved to the next tube for image collection. Each image viewed an area of 18 mm × 13.5 mm at a resolution of 640 pixels × 480 pixels. Acquired images had a redundant frame that was cropped, resulting in images with a size of 624 pixels × 450 pixels.

#### Automated image acquisition

Automated image acquisition was performed using the integrated system RootCam (CrystalVision, Samar, Israel) with its default settings. RootCam includes the camera itself and software designed to move the camera along a rail to acquire images every 18.75 mm along a plant root. Lighting was supplied by light-emitting diode strips. The images were saved to a “Raspberry Pi” device, which was accessible via a network cable and allowed the image acquisition time intervals to be set with remote control. Each image viewed an area of 25 mm × 19 mm with a resolution of 2,952 pixels × 1,944 pixels.

**Fig. 4.**
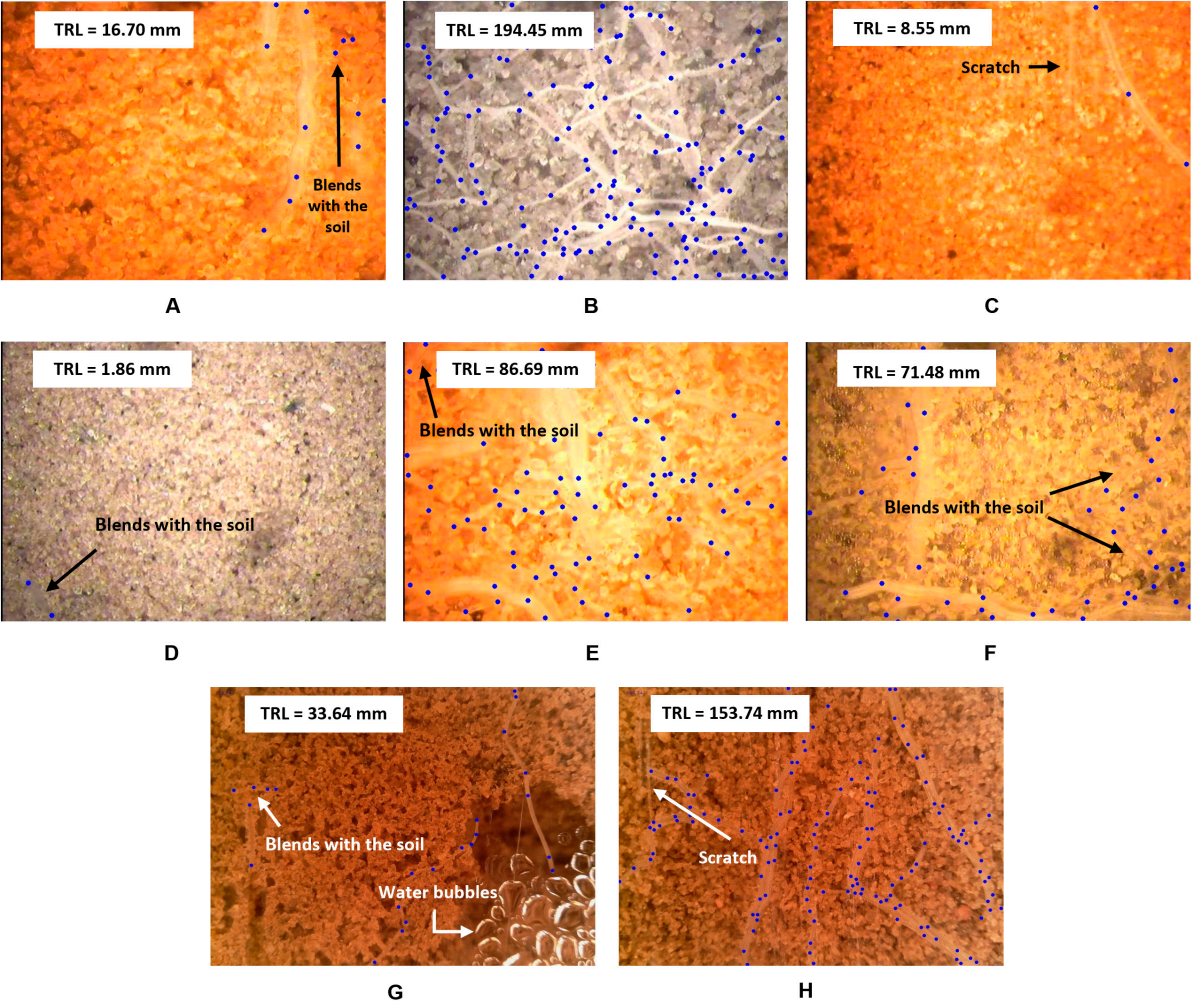
Examples of images with GT point annotations (blue points, drawn on top) derived from Rootfly and the GT TRL values (in millimeters). The images demonstrate the different difficulties in handling such images and are of different crops and different acquisition systems: (A and B) corn, (C and D) melon, (E and F) tomato, and (G and H) pepper, where images (A) to (F) were acquired with the manual system and images (G) and (H) with the automated system. These difficulties include the differences when (C and D) a single root or (B) multiple roots are present in an image; (A to H) inconsistent scene illumination between images, even of the same crop; (A and D to G) low contrast between roots and soil; and artifacts, such as (C and H) scratches that may look like roots or (G) water bubbles.

Analysis of the root images captured by the MR systems was challenging since they may have been blurry or had poor contrast between the roots and the background. Moreover, the background of some images contained artifacts such as scratches, stains, and water bubbles on the observation tubes. However, these challenges are more prominent in the manual MR system because of its inconsistent illumination and lower resolution that caused poorer quality of the obtained images. These had more roots “blended” with the soil than images obtained with the automated system. Therefore, the datasets acquired with the manual MR system were more challenging even for a human annotator. Because of the substantial differences in image quality, the datasets are distinguished on the basis of the acquisition system and are referred to as different dataset types. Examples of these challenges, from both the manual and automated acquisition systems, can be seen in Fig. 4, in which the annotations of the GT points derived from Rootfly are given on top of each root.

### Datasets

Using the 2 acquisition systems, 4 datasets (denoted datasets 1, 2, 3, and 4) were obtained from 6 different experiments (Table S2). The experiments included 4 different crop species (corn, pepper, melon, and tomato) grown under stressed conditions (drought, nitrogen deficiency, low temperature, salt stress, or salt stress combined with container-induced root restriction) in different years (designated “Melon 2018”, “Melon 2019”, “Tomato 2019”, “Tomato 2020”, “Corn 2020”, and “Pepper 2021”). The soil used for “Pepper 2021” was loamy sand, and the soil used for the other experiments was sand (Table S3).

The images of datasets 1 and 2 were randomly chosen from different tubes, locations (soil depths), and dates of measurement and were used for both training and testing the models. Additional datasets, datasets 3 and 4, were obtained to further test the trained models and to generate RLD curves. To do so, each of the datasets used for the estimation of RLD included time-series images acquired from the same tube at different locations. Since the acquisition protocol for these additional datasets was not randomized by crop, tube, location, or time, they were not used for training.

• Dataset 1 included images from all 6 experiments, initially with 200 images per experiment, which were randomly selected. After 57 extremely corrupted images were deleted, this was manually determined, and the data were randomly divided into training, validation, and test sets, with 73%, 9%, and 18% of the data in each set, respectively. This resulted in 836 images for training, 104 for validation, and 203 for testing.
• Dataset 2 included 420 images acquired using the automated MR system, randomly chosen from the “Pepper 2021” experiment. The set was randomly split such that 72% was used for training, 10% for validation, and 18% for testing.
• Dataset 3 included 832 images from the “Pepper 2021” experiment, acquired using the automated MR system in a time series, at intervals of 5 d. It was only used for testing.
• Dataset 4 included a total of 2,452 images acquired using the manual MR system from all 6 experiments. The time series of root images in each experiment was acquired using the manual MR system, at intervals of 14 d after planting. It was only used for testing.

#### Ground truth

GT was obtained with manual annotations using the Rootfly software, in which the user adds points along the selected root. These points usually correspond to the coordinates at the start and the end of the root, the splitting points between the main root and other roots, and points to describe the curvature of the root. These points are then connected in a line, the length of which reflects the real length of the selected root. This is done for all roots within an image and for all images in a dataset. While “start”, “end”, and “split points” are well defined, points to describe the curvature of the root cannot be precisely defined and essentially, and 2 people would annotate different points. This means that by definition, a network cannot detect such points with high accuracy (because the arbitrary points along the root are not detectable, they do not have any defining criterion). This, in turn, does not mean that the points model cannot accurately estimate the TRL (the network learns to estimate it from arbitrarily detected root points) and to infer (approximately) the sum of distances between near points.

### Sensitivity analysis—Creating a subset of dataset 1

To examine the influence of the dataset size and MR system type (manual or automated), an additional subset of images was randomly chosen from dataset 1, with a similar size to dataset 2 (including the same numbers of images with and without roots), for training, validation, and testing. This allowed us to compare the 2 different MR systems without the effect of sample size.

### Evaluation

#### Metrics

To evaluate the metrics of the models, *y_i_* was defined as observation *i* (true TRL value of image *i*), $\hat{y_{i}}$ as the automated estimation of TRL for image *i*, *n* as the sample size (number of images), and $\bar{y}$ as the mean of all *n* (true) observations.

The results were evaluated using the coefficient of determination, denoted *R*^2^, between the GT manual annotation (dependent variable) and model’s estimated TRL values and the following metrics:

1. ∣∆*RL*∣: The mean of the absolute difference between the GT value and model’s estimate for TRL per image (Eq. 1).

$$\left|∆\mathrm{RL}\right|=\frac{1}{n}\sum_{i=1}^{n}\left|y_{i}-\hat{y_{i}}\right|$$ (1)

2. Mean relative deviation (MRD): The average relative error of the model’s estimates (Eq. 2). This is only relevant to images with roots.

$$\text{MRD}=\frac{1}{n}\sum_{i=1}^{n}\frac{\left|y_{i}-\hat{y_{i}}\right|}{y_{i}}$$ (2)

3. Normalized root mean square error (NRMSE) (Eq. 3).

$$\text{NRMSE = }\frac{\sqrt{\sum_{i=1}^{n}\frac{{\left(y_{i}-\hat{y_{l}}\right)}^{2}}{n}}}{y_{\text{max}}-y_{\text{min}}}$$ (3)

4. Fraction of explained variance, 1 − fraction of variance unexplained (FVU) (Eq. 4)*.*

$$1-\mathrm{FVU}=1-\frac{\sum_{i=1}^{n}{\left(y_{i}-\hat{y_{i}}\right)}^{2}}{\sum_{i=1}^{n}{\left(y_{i}-\bar{y}\right)}^{2}}$$ (4)

This statistic is used to examine whether the suggested models’ performance (its mean square error is the nominator) is preferable than always estimating TRL with the trivial value of mean *y_i_* (the mean square error of this estimator is the denominator).

#### Analysis

Each model was trained with 300 epochs, using the relevant training set. The relevant validation set was used to choose the best epoch based on the MRD values obtained for it. The trained model with chosen weights was then tested on the test set of the relevant dataset (the results are presented in the “Results” section). The following cases were evaluated:

1. The suggested models’ performance and comparison to previous segmentation-based models. For each model, training and testing were conducted on the same dataset type (using the training, validation, and test sets of datasets 1 and 2). The presented results are compared with previously reported published studies [16,19] that performed automated TRL estimation with a segmentation-based model.
2. Image quality and dataset size—sensitivity analysis results. The effects of dataset size and image quality were examined by comparing the results obtained using datasets 1 and 2 with these obtained using the subset of dataset 1 that was the same size as dataset 2.
3. Binary classification. Automatic distinguishment between images with roots from those without roots. The classification was based on the 2 models after they were trained using dataset 1 (the largest set). To output the images that had no roots in the test set, a threshold was chosen for the TRL estimate of the model. For images in the test set with estimated TRLs smaller than this threshold, the binary output was “no roots”; otherwise, the output was “has roots”. The threshold for both models was determined by selecting the estimated TRL value at the 80th percentile of the “no roots” images in the validation set of dataset 1. The images in the validation set without roots and with an estimated TRL value below the chosen threshold had a TRL estimate of 0 for both models.
4. Comparing root traits with RLD calculations. The trained models were tested on datasets 3 and 4, and RLD plots were generated to compare the outputs on the basis of the manual annotations versus the models’ estimates. Since the MR technique forms images in 2 dimensions, the RLD was calculated from the TRL for the specific imaging area (in centimeters per square centimeter). Data preparation and graphics were conducted with the packages “dplyr”, “reshape2”, and “ggplot2” in RStudio Desktop (version 1.4.1725) software with R (version 4.1.1).
5. Comparison with a joint model—using both dataset types for training**.** The points model was trained and tested by joining both types of images, those that were obtained with the manual MR system and those obtained with the automated MR system. This was done by combining the training, validation, and test sets of datasets 1 and 2.
6. Examining the transfer learning possibilities. The effect of the dataset type used for training a model on the performance of that model when tested on a different type of data was evaluated. This was examined by first presenting the test images for dataset 2 to a model trained exclusively on dataset 1, and vice versa. Second, evaluating how fine-tuning the model by introducing additional training images from the other data type improved the results. This was demonstrated using a model originally trained on dataset 2, which was then fine-tuned by additional training with a subset of images randomly chosen from the training set of dataset 1. The effect was examined as a function of the additional subset size by varying the number of additional training images in the range of 10 to 200. In the preliminary examination, it was observed that a model could perform well on another dataset type for images without roots. Therefore, the focus here was specifically on adding images with roots for the additional training.

#### Computation

All experiments were conducted using AMD Ryzen 2920X CPU, NVIDIA GeForce RTX 2080 Ti graphics processing unit (GPU), CUDA 11.3, and PyTorch 1.2. TRL estimation took <1 s per image using a GPU in test time.

## Results

### Training and testing with the same dataset type

#### The suggested models’ performance and comparison to previous segmentation-based models

Results revealed that both suggested models (the regression model and points model) achieve higher *R*^2^ values than Smith et al. [16] and Bauer et al. [19], whereas the NRMSE values of Bauer et al. [19] were in the same range (Table 1).

**Table 1.** Evaluation metrics for the estimation of TRL using data from both the manual and automated MR cameras, when trained and tested on the same dataset type using the regression and points models. The results compared to previous works of automated root length estimations. As part of the sensitivity analysis, the results for the subset of dataset 1 that was the same size as dataset 2 are presented.

Model	Dataset	∣∆*RL*∣	MRD (GT > 0)	NRMSE	*R* ^2^	1 − FVU
Smith et al. [16]	Referred in [16]	-	-	-	0.890–0.920	-
Bauer et al. [19]	Referred in [19]	-	-	0.039–0.077	0.590–0.810	-
Regression model	Dataset 1	4.50	28.2%	0.056	0.929	0.90
	Dataset 2	4.90	10.7%	0.034	0.981	0.97
	Dataset 1–subset	5.95	29.0%	0.071	0.875	0.86
Points model	Dataset 1	3.19	20.9%	0.041	0.958	0.95
	Dataset 2	4.88	10.6%	0.030	0.986	0.98
	Dataset 1–subset	5.04	29.2%	0.053	0.923	0.92

Results (Table 1) show that the points model outperformed the regression model in all cases on all metrics (except the MRD values for the subset of dataset 1, which differed by 0.16% in favor of the regression model). When tested on dataset 2, the differences between the models were very small (e.g., a difference of 0.1% in MRD and 0.005 in the *R*^2^ value). In contrast, for dataset 1, which was the more challenging dataset, the points model showed an advantage in terms of the average estimation error per image, with an MRD value lower by 7.3% than that of the regression model. This implies that having high-quality images (dataset 2) allowed the automated estimation of TRL values with MRD values of ~10%, even without information about the point coordinates, when the regression model was used. However, with a more challenging dataset such as dataset 1, the points model, which incorporates additional information about the root (point) coordinates, produced substantially better results, reducing the MRD results from ~28% to ~21%.

#### Image quality and dataset size—Sensitivity analysis results

A comparison of the results for the same model on different dataset types (dataset 1 versus dataset 2) revealed that for both models, better results were obtained with the automated MR system images (dataset 2) on all metrics except NRMSE. The MRD values were lower by 17.5% and 10.3% for dataset 2 than for dataset 1 with the regression model and points model, respectively, although dataset 2 was more than 2-fold smaller. This implies that image quality is a major influence on model performance. To determine the influence of the dataset type (and thus the type of image acquisition system), the results of dataset 2 were compared with those obtained for the subset of dataset 1 (which was the same size as dataset 2). Increasing the dataset size (using the full dataset 1) improved the results compared with those achieved using its subset, but the results for dataset 2 remained considerably better than those for dataset 1. Although the results for dataset 2 remained better with both models, the combination of the larger dataset (full dataset 1) with additional information about the root point coordinates (when using the points model) yielded improved results for the subset of dataset 1. The improvement due to this increase in dataset size was greater in the points model, in which the MRD value decreased from 29.2% to 20.9%, whereas in the regression model, the MRD value decreased by only 0.84%. Therefore, with more-challenging data, using the points model and increasing the dataset size improved the TRL estimation.

Since the points model proves to be more robust to changes in image quality, the results of the next sections are based on this model.

#### Binary classification—Distinguishing between images with and without roots

**Fig. 5.**
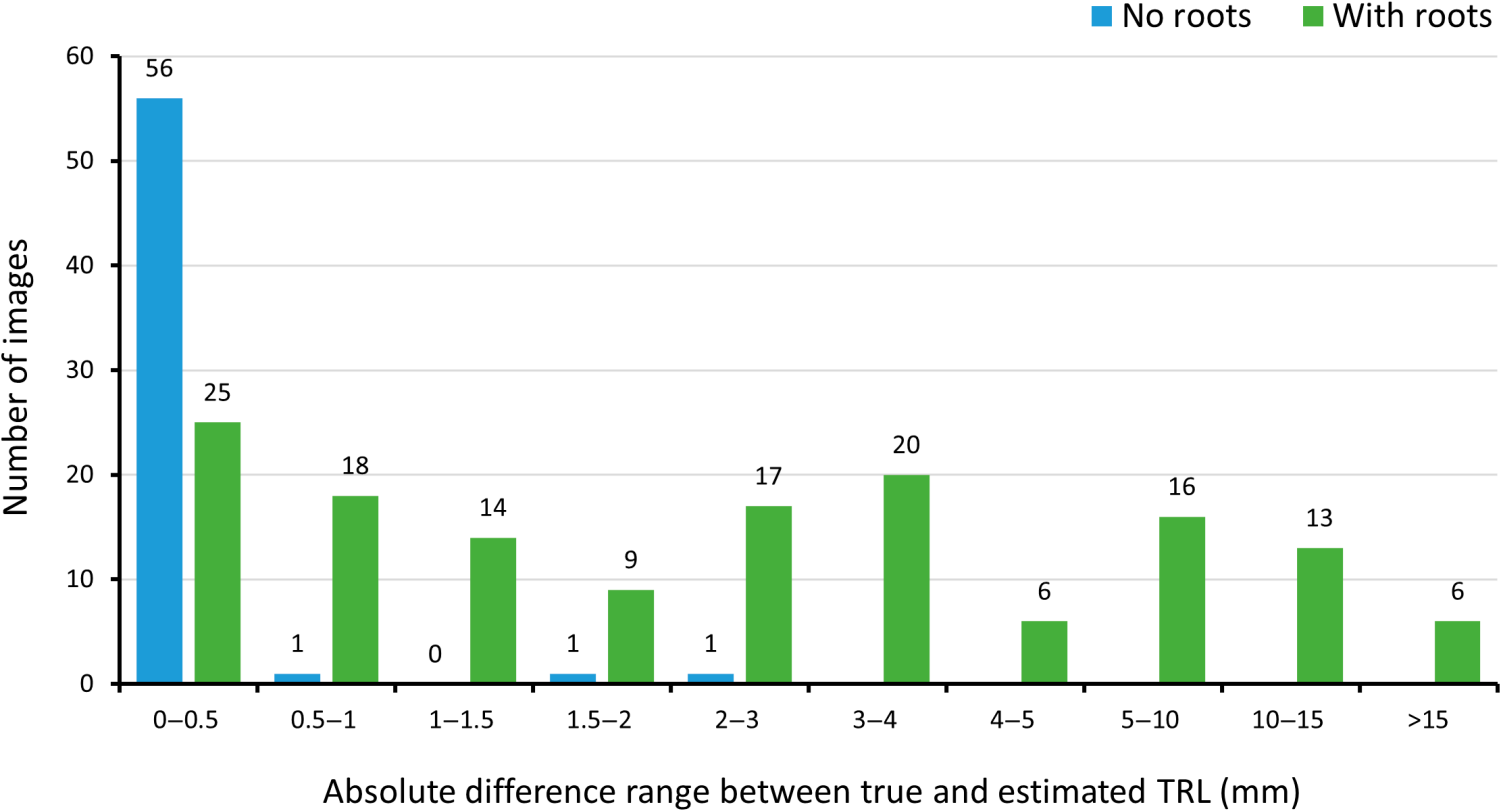
The number of images in each range of absolute differences between manual annotations of TRL values and the points model estimates for the test set of dataset 1. Blue bars show the results for images with roots, and red bars show those for images without roots.

The |Δ*RL*| values are of specific interest for images without roots. This will be demonstrated for dataset 1 (the largest dataset). Each image in this dataset was classified in either the “no roots” or the “has roots” category based on a preset threshold for |Δ*RL*|. For the points model, the threshold value for deciding whether an image had roots based on the validation set was 0.37 mm. This threshold resulted in a 3% error for this binary task. For the regression model, the threshold was set to 0.027 mm, and the resulting error on the test set was 5%. Figure 5 presents the |Δ*RL*| values for the test set of dataset 1 when the points model was used, which included 144 images with roots and 59 images without roots (Table S2). It can be seen from these results that 57 of the 59 images (~97%) without roots had an estimated TRL of <1 mm, indicating that the points model is robust in detecting images without roots.

#### Comparing root traits with RLD calculations

**Fig. 6.**
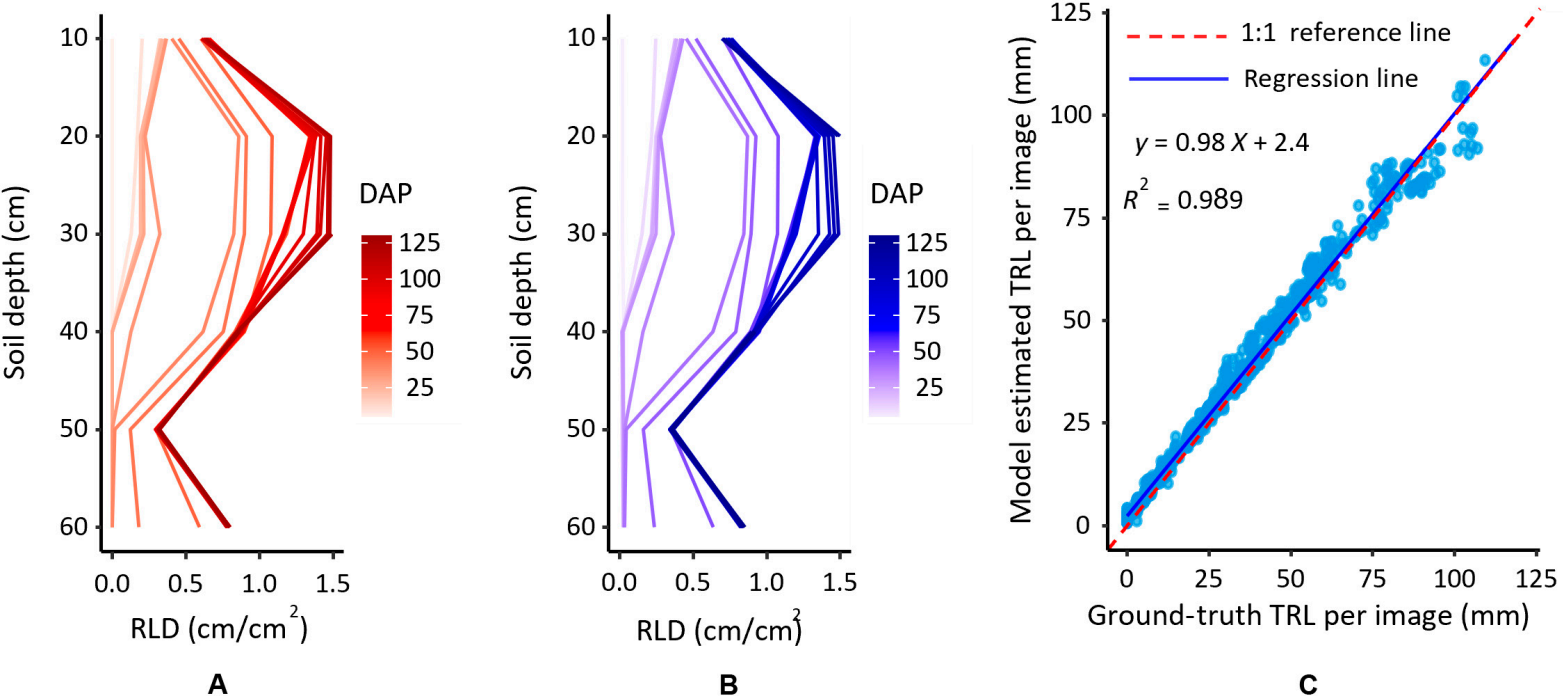
Comparison of RLD with depth for dataset 3 analyzed using (A) manual annotation and (B) model estimation. (C) Correlation plot for the points model when tested on dataset 3. DAP, days after planting. The red dashed line and blue solid line represent the 1:1 reference line and the regression line, respectively. The blue circle dots represent TRL values per image from testing dataset.

**Fig. 7.**
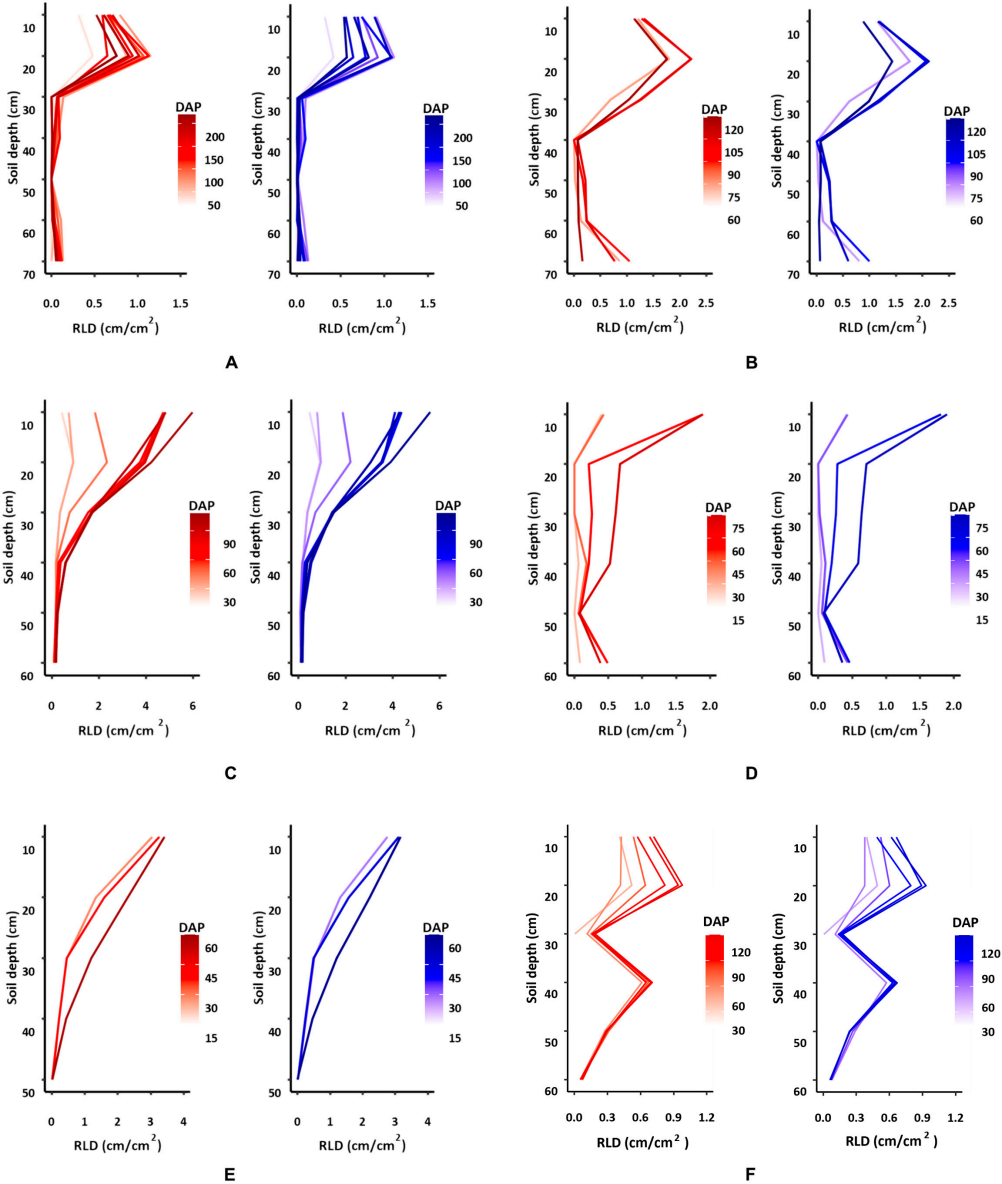
Comparison of RLD calculated from images captured on different days by manual annotation using Rootfly (red line on the left side) or by model estimation (blue line on the right side) in 6 experiments when tested with the points model on dataset 4: (A) Melon 2018, (B) Melon 2019, (C) Tomato 2019, (D) Tomato 2020, (E) Corn 2020, and (F) Pepper 2021.

One specific parameter addressed in this work, which is based on TRL estimates, is the total length of roots per unit of the observed area (RLD). It is an indicator of root distribution in the soil, which is important in understanding the extraction of water and nutrients from the soil [51]. Therefore, RLD can be used to evaluate the response of roots to various environmental conditions [52–55]. The comparison of the RLD calculations based on data generated from manual annotations (GT values) and those generated from the points model TRL estimates for dataset 3 (Fig. 6A and B) and dataset 4 (Fig. 7) demonstrate similar patterns for the GT values and the model’s estimates. It is important to note that similar patterns were obtained for images acquired by both MR systems, the automated (dataset 3) and manual (dataset 4), despite the substantial differences in image quality.

**Fig. 8.**
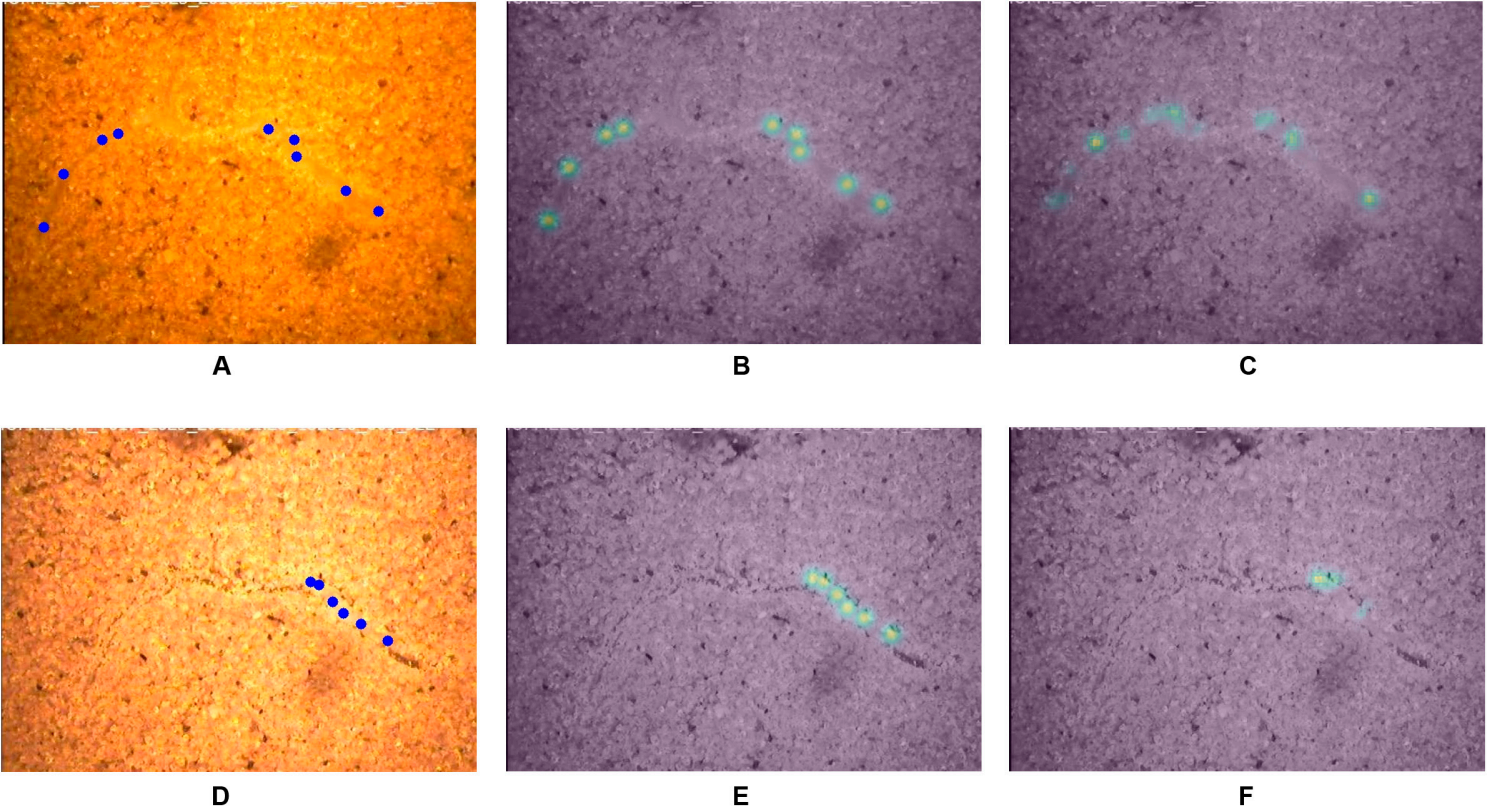
Examples displaying the models' ability to recognize root disappearance. In the first row, there are (A) one raw image acquired from the “Melon 2018” experiment with GT points annotations made in Rootfly drawn on top of it, (B) GT heatmap based on Rootfly annotations on top of the raw image, and (C) model estimation of the point heatmap for this image. In the second row, there are (D) the raw image from the same location taken after 50 d with GT points annotations made in Rootfly drawn on top of it, (E) GT heatmap based on Rootfly annotations on top of the raw image, and (F) model estimation of the points heatmap for this image.

The root traits results in Figs. 6 and 7 are important to farmers when deciding the amounts of water and fertilizer to apply on the basis of root exploration and real-time root dynamics. Furthermore, the reduced RLD at entire soil depths at later measurement dates on “Melon 2018” and “Melon 2019” data (Fig. 7A and B) shows that the points model successfully identified “root disappearance”. This is also demonstrated by the points heatmap generated by the model (Fig. 8) and indicates that the proposed model can track the appearance, growth, and disappearance of individual roots over time, allowing the accurate estimation of root production and turnover. This is important when studying the permanent disappearance of fine roots, which is a critical criterion for the transition from dead root to soil organic matter via decomposition [56–59]. Specifically, this model can detect either the loss of length from existing roots or the complete disappearance of a root(s), which indicates that the model can achieve human-expert-level performance.

Table 2 presents the metrics results when the points model was tested on datasets 3 and 4. Although the error values (15.2%) for dataset 3 are larger than those for the test set of dataset 2 (10.6%; Table 1), the correlation between the GT values and the model’s estimates remains very high (*R*^2^ = 0.989; Fig. 6C and Table 2).

**Table 2.** Results of the points model when tested on dataset 3 (automated MR camera) and dataset 4 (manual MR camera).

Test set	Experiment	∣∆*RL*∣	MRD (GT > 0)	NRMSE	*R* ^2^	1 − FVU
Dataset 3	Pepper 2021	2.61	15.2%	0.031	0.989	0.98
Dataset 4	Melon 2018	1.63	35.7%	0.062	0.908	0.90
	Melon 2019	2.37	17.4%	0.049	0.967	0.96
	Tomato 2019	3.81	21.4%	0.036	0.987	0.98
	Tomato 2020	1.23	18.3%	0.025	0.977	0.98
	Corn 2020	2.87	18.9%	0.030	0.986	0.98
	Pepper 2021	1.49	23.7%	0.046	0.961	0.96

Table 2 also presents the results when the points model was tested on dataset 4. For 5 of the 6 experiments in dataset 4 (all except “Melon 2018”), the results were close to the MRD obtained for the test set of dataset 1 (20.9%; Table 1), with higher correlation values. Three of these experiments had better results than those obtained for dataset 1 (“Melon 2019”, “Tomato 2020”, and “Corn 2020”), but the results of one experiment (“Melon 2018”) were an exception, with a ~15% increase in MRD relative to the dataset 1 results. These experiments’ images were especially challenging because of the illumination issue and the extreme blending of the roots with the color of the soil.

### Comparison with a joint model—Using both dataset types for training

The “joint model” refers to the points model when trained and evaluated using both dataset 1 and dataset 2. The results (Table 3) reveal that the model can handle a variety of root images while training, and, in fact, it performed as well as it did when trained and tested on the same type of images. It even performed slightly better with a mix of image types during training, with MRD values decreasing by 0.7% for the test set of dataset 1 and by 1.1% for dataset 2, and the *R*^2^ values increasing by 0.008 and 0.003 (i.e., remaining almost the same), respectively. The fact that training with more images and different types of images (from both manual and automated systems) provides an enhanced result implies that if multiple dataset types are available for training, it is better to join the datasets for training instead of training each dataset individually. The larger and more varied the training set, the better the model will perform in testing.

**Table 3.** Test results for the points model trained on both dataset types, manual and automated MR camera (datasets 1 and 2). The results of the model when trained only on the same dataset type as the test set, taken from Table 1, are given in parentheses.

Training and validation set	Test set	∣∆*RL*∣	MRD (GT > 0)	NRMSE	*R* ^2^	1 − FVU
Joint model (datasets 1 + 2)	Joined model (datasets 1 + 2)	3.53	16.7%	0.029	0.980	0.97
	Dataset 1	3.00 (3.19)	20.2% (20.9%)	0.035 (0.041)	0.966 (0.958)	0.96 (0.95)
	Dataset 2	4.92 (4.88)	9.7% (10.6%)	0.034 (0.030)	0.989 (0.986)	0.97 (0.98)

### Examining the transfer learning possibilities

**Fig. 9.**
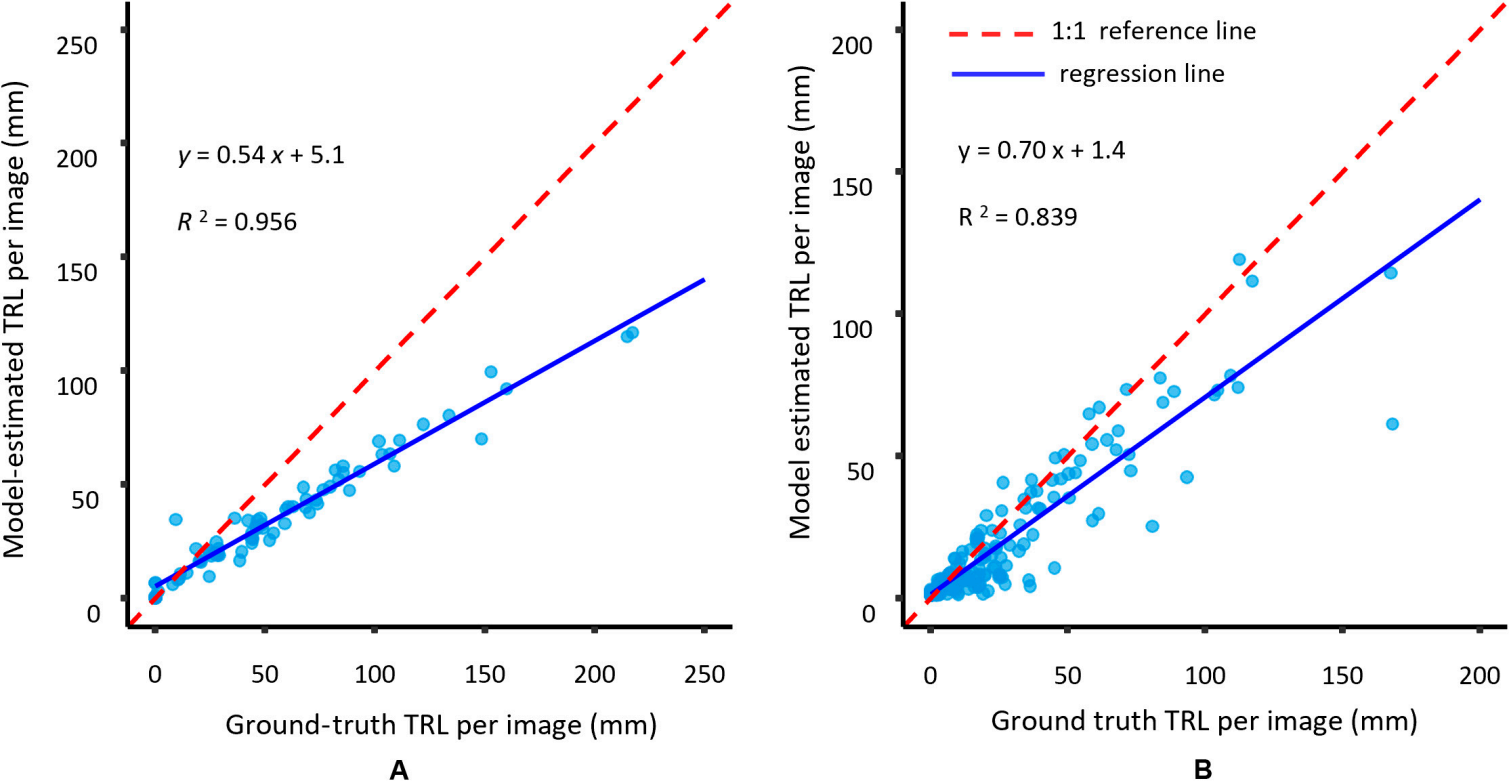
Correlation plots between GT TRL per image generated by manual annotation and the estimated TRL per image generated using the points model when (A) trained on dataset 1 and tested on dataset 2 and (B) trained on dataset 2 and tested on dataset 1. The red dashed line and blue solid line represent the 1:1 reference line and the regression line, respectively. The blue circle dots represent TRL values per image from testing dataset.

Transfer learning is examined by testing a trained model on another data type and fine-tuning by allowing additional training. The results of testing the points model on different data types than the type upon which it was trained (manual versus automated system) revealed that the greatest decline in performance occurred when a model that was trained with automated MR system data (dataset 2) was tested on new data from a manual MR system (dataset 1) (Table 4 and Fig. 9). This resulted in an MRD of 39.0% and *R*^2^ of 0.839 (Table 4) compared with 20.9% and 0.958, respectively, when trained on dataset 1 (Table 1).

**Table 4.** Results of TRL estimation using the points model when tested on a dataset that differed from the training dataset.

Training and validation set	Testing set	∣∆*RL*∣	MRD (GT > 0)	NRMSE	*R* ^2^	1 − FVU
Dataset 1 (manual MR system)	Dataset 2 (automated MR system)	21.43	38.6%	0.137	0.956	0.58
Dataset 2 (automated MR system)	Dataset 1 (manual MR system)	7.45	39.0%	0.083	0.839	0.78

This means that training on lower-quality data (dataset 1) and testing on high-quality data (dataset 2) gave better results than the opposite situation. This is probably attributable to 3 factors. The first is that dataset 1 is almost twice as large as dataset 2, and the bigger the training set, the better the generalizability of the model to new types of images. Second, the images in dataset 1 are more diverse because of their poorer quality than the images from the automated system (dataset 2), which also allows better generalizability. Third, testing a model on more-challenging images when the model was trained on “easier” images also contributed to the reduction in performance on more-difficult data (e.g., in dataset 1, the roots are more difficult to see because their color, more often, blends with the soil). Figure 9 also shows a consistent and proportional bias between model estimation and manual annotation, indicating a systematic underestimation of TRL with model estimation. Greater bias was observed (Fig. 9A) when the model was trained on images taken with the manual MR system (dataset 1) and tested on images with the automated MR camera (dataset 2). Furthermore, the more roots present in the images, the more likely was this error (Fig. 9). Although deterioration in performance was observed when the model was tested on images taken from a different MR system, the bias in the method was systematic and very predictable given the high correlation value.

In the next step, an attempt was made to improve results for the case that had a greater performance decline (model trained on dataset 2 and tested on dataset 1). The transfer learning options were examined using this model by performing additional training of the given model with datasets of increasing size from dataset 1. This approach also quantified the additional annotations that were required when an existing trained model was used on a new type of data. The results of these additional training options (Table S4) revealed that by adding only 10 additional training images from another dataset, the model’s performance was considerably improved: MRD decreased from 39.0% to 31.3% and *R*^2^ improved from ~0.839 to ~0.927. However, adding further training images improves the results at a slower rate as an increasing number of images are required to get closer to the original performance of a model that was trained and tested upon the same data type.

## Discussion

Two CNN models that were previously developed for a part counting task were adapted to automatically analyze root images providing a method for accurately analyzing large numbers of MR images without need of prior segmentation. Results revealed MRD values of 20.9% and 10.6% for the manual and automated MR systems, respectively. The specific contributions of this work are as follows:

1. Automated tools for estimating TRL from MR images are presented, the results of which correlate strongly with manually obtained values: *R*^2^= 0.929 to 0.958 for images from the manual MR system (dataset 1) and *R*^2^= 0.981 to 0.989 for images from the automated MR system (dataset 2). These strong correlations of both models may allow understanding of real-time root growth dynamics, particularly in response to various environmental factors. It also facilitates informed decision-making processes with high accuracy.
2. We contribute to future R&D in root image analysis by making publicly available dataset of MR images with thoroughly examined root annotations.
3. The points model, which identifies the coordinates of roots in an image (outputs an estimate of the heatmap of points coordinates along the roots), allows the visual inspection of the roots in each image and the examination of the human annotations. This visual inspection feature could reduce errors in annotations, caused by human inconsistency and subjectiveness, leading to improved annotation quality.
4. The ability of the suggested regression model to accurately estimate TRL values per MR image is demonstrated. This is particularly important as it enables training a model capable of accurate automated analysis without requiring any annotations apart from the TRL value.
5. The models can be used to distinguish between images with and without roots. This was done by setting a threshold to the TRL estimate, and in the case of an estimate smaller than this threshold, the binary output was “no roots”; otherwise, the output was “has roots”. This resulted in 3% and 5% errors for this binary task for the points model and the regression model, respectively. This allows accurate monitoring of the appearance and disappearance of individual roots over time.
6. The ability of a CNN model trained to estimate TRL values on a specific dataset to analyze new datasets (new distributions) is demonstrated. The change in performance is quantified and the ability of the points model to improve as a function of the number of additional training images from the new dataset is demonstrated. It was shown that as few as 10 additional training images can offer a substantial improvement. This implies that using a small sample from a new dataset (even if it has more “difficult” images) for additional training can enhance the model’s performance, confirming the generalizability of the model. Dozens of additional training images, rather than thousands, are required. This is important, especially for root studies, where datasets tend to be relatively small, and annotation is time-consuming.

We recommend the following guidelines to improve TRL estimation:

• If possible, train with more images and different types of images (from both manual and automated systems). If multiple dataset types are available for training, it is better to join the datasets for training instead of training each dataset individually. The larger and more varied the training set, the better the model will perform in testing.
• If data consist of both lower- and higher-quality images, including lower-quality data in the training are beneficial for achieving better accuracy.
• Transfer learning—if a trained model is available, it is important to use some of the new annotated images for validation before evaluating the model on the new data. To improve the model’s accuracy, additional training should be done incrementally to save on annotation costs until satisfying accuracy or a plateau on the validation set is obtained.

## Conclusion

This study provides automated tools for estimating TRL from MR images without requiring segmentation. This method can enable rapid and reliable estimation of root growth patterns, allowing root phenotyping and analysis with high temporal resolution. The proposed model can track the appearance, growth, and disappearance of individual roots over time, allowing the accurate estimation of root production and turnover. This will help growers to make sound decisions about water and nutrient supply based on up-to-date root growth information and to predict plant stress before visible stress symptoms appear. This has important implications for the timely and cost-effective control of stress in precision agriculture. The proposed approach can potentially maximize the utilization of MR-based root phenotyping platforms. This can also be extended to images undergoing super-resolution procedures, aiming to enhance image resolution.

Ongoing research is focused on using these models to estimate additional aggregated properties of roots, such as mean root diameter in an image. In such a case, the mean diameter will be the learned GT value instead of TRL. Moreover, these models can be utilized to analyze individual root images, extracted (i.e., cropped) from larger MR images, enabling estimation of per-root diameter or color. These properties, in turn, can facilitate the classification or differentiation of various root types. For instance, categorizing roots into fine and coarse categories based on their diameters or establishing age-related classifications (e.g., young versus old) based on root color.

Expanding upon these methodologies, further directions could involve employing the suggested models to estimate intricate details like root hairs’ quantity, length, or diameter.

## Data Availability

The datasets generated and analyzed during the current study are available in the Zenodo repository, https://doi.org/10.5281/zenodo.7482146.

## References

[1] Molotoks A, Smith P, Dawson TP. Impacts of land use, population, and climate change on global food security. Food Energy Secur. 2021;10(1): Article e261.

[2] Koevoets IT, Venema JH, Elzenga JTM, Testerink C. Roots withstanding their environment: Exploiting root system architecture responses to abiotic stress to improve crop tolerance. Front Plant Sci. 2016;7:1335. 27630659 10.3389/fpls.2016.01335 PMC5005332

[3] Lynch JP. Rightsizing root phenotypes for drought resistance. J Exp Bot. 2018;69(13):3279–3292. 29471525 10.1093/jxb/ery048

[4] Lynch JP, Strock CF, Schneider HM, Sidhu JS, Ajmera I, Galindo-Castañeda T, Klein SP, Hanlon MT. Root anatomy and soil resource capture. Plant Soil. 2021;466(1):21–63.

[5] Ajmera I, Henry A, Radanielson AM, Klein SP, Ianevski A, Bennett MJ, Band LR, Lynch JP. Integrated root phenotypes for improved rice performance under low nitrogen availability. Plant Cell Environ. 2022;45(3):805–822. 35141925 10.1111/pce.14284 PMC9303783

[6] Amtmann A, Bennett MJ, Henry A. Root phenotypes for the future. Plant Cell Environ. 2022;45(3):595–601. 35092061 10.1111/pce.14269

[7] Ephrath JE, Klein T, Sharp RE, Lazarovitch N. Exposing the hidden half: Root research at the forefront of science. Plant Soil. 2020;447(1):1–5.

[8] Falk KG, Jubery TZ, Mirnezami SV, Parmley KA, Sarkar S, Singh A, Singh AK. Computer vision and machine learning enabled soybean root phenotyping pipeline. Plant Methods. 2020;16(1):1–19. 31993072 10.1186/s13007-019-0550-5 PMC6977263

[9] McGrail RK, Van Sanford DA, McNear DH Jr. Trait-based root phenotyping as a necessary tool for crop selection and improvement. Agronomy. 2020;10(9):1328.

[10] Hartmann A, Šimůnek J, Aidoo MK, Seidel SJ, Lazarovitch N. Implementation and application of a root growth module in HYDRUS. Vadose Zone J. 2018;17(1):1–16.

[11] Kuppe CW, Kirk GJD, Wissuwa M, Postma JA. Rice increases phosphorus uptake in strongly sorbing soils by intra-root facilitation. Plant Cell Environ. 2022;45(3):884–899. 35137976 10.1111/pce.14285

[12] Lak ZA, Sandén H, Mayer M, Godbold DL, Rewald B. Plasticity of root traits under competition for a nutrient-rich patch depends on tree species and possesses a large congruency between intra-and interspecific situations. Forests. 2020;11(5):528.

[13] Zhu J, Ingram PA, Benfey PN, Elich T. From lab to field, new approaches to phenotyping root system architecture. Curr Opin Plant Biol. 2011;14(3):310–317. 21530367 10.1016/j.pbi.2011.03.020

[14] Tracy SR, Nagel KA, Postma JA, Fassbender H, Wasson A, Watt M. Crop improvement from phenotyping roots: Highlights reveal expanding opportunities. Trends Plant Sci. 2020;25(1):105–118. 31806535 10.1016/j.tplants.2019.10.015

[15] Rewald B, Ephrath JE. Minirhizotron techniques. In: Plant roots: The hidden half.Fourth. Boca Raton (FL): CRC Press; 2013. pp. 735–750.

[16] Smith AG, Han E, Petersen J, Olsen NAF, Giese C, Athmann M, Dresbøll DB, Thorup-Kristensen K. RootPainter: Deep learning segmentation of biological images with corrective annotation. New Phytol. 2022;236(2):774–791. 35851958 10.1111/nph.18387 PMC9804377

[17] Danilevicz MF, Bayer PE, Nestor BJ, Bennamoun M, Edwards D. Resources for image-based high-throughput phenotyping in crops and data sharing challenges. Plant Physiol. 2021;187(2):699–715. 34608963 10.1093/plphys/kiab301 PMC8561249

[18] Zeng G, Birchfield ST, Wells CE. Automatic discrimination of fine roots in minirhizotron images. New Phytol. 2008;177(2):549–557. 18042202 10.1111/j.1469-8137.2007.02271.x

[19] Bauer FM, Lärm L, Morandage S, Lobet G, Vanderborght J, Vereecken H, Schnepf A. Development and validation of a deep learning based automated minirhizotron image analysis pipeline. Plant Phenomics. 2022;2022:9758532. 35693120 10.34133/2022/9758532 PMC9168891

[20] LeCun Y, Bengio Y, Hinton G. Deep learning. Nature. 2015;521(7553):436–444. 26017442 10.1038/nature14539

[21] Jiang Y, Li C. Convolutional neural networks for image-based high-throughput plant phenotyping: A review. Plant Phenomics. 2020;2020:4152816. 33313554 10.34133/2020/4152816 PMC7706326

[22] Li Z, Guo R, Li M, Chen Y, Li G. A review of computer vision technologies for plant phenotyping. Comput Electron Agric. 2020;176: Article 105672.

[23] Pound MP, Atkinson JA, Townsend AJ, Wilson MH, Griffiths M, Jackson AS, Bulat A, Tzimiropoulos G, Wells DM, Murchie EH, et al. Deep machine learning provides state-of-the-art performance in image-based plant phenotyping. Gigascience. 2017;6(10):gix083. 10.1093/gigascience/gix083 PMC5632296 29020747

[24] Singh AK, Ganapathysubramanian B, Sarkar S, Singh A. Deep learning for plant stress phenotyping: Trends and future perspectives. Trends Plant Sci. 2018;23(10):883–898. 30104148 10.1016/j.tplants.2018.07.004

[25] Fajardo M, Whelan BM. Within-farm wheat yield forecasting incorporating off-farm information. Precis Agric. 2021;22(2):569–585.

[26] Saleem MH, Potgieter J, Arif KM. Automation in agriculture by machine and deep learning techniques: A review of recent developments. Precis Agric. 2021;22(6):2053–2091.

[27] Castro-Valdecantos P, Apolo-Apolo OE, Pérez-Ruiz M, Egea G. Leaf area index estimations by deep learning models using RGB images and data fusion in maize. Precis Agric. 2022;23:1949–1966.

[28] Farjon G, Krikeb O, Hillel AB, Alchanatis V. Detection and counting of flowers on apple trees for better chemical thinning decisions. Precis Agric. 2020;21(3):503–521.

[29] Kalantar A, Dashuta A, Edan Y, Dafna A, Gur A, Klapp I. Estimating melon yield for breeding processes by machine-vision processing of UAV images. In: Precision agriculture’19. Wageningen (The Netherlands): Wageningen Academic Publishers; 2019. pp. 1386–1393.

[30] Koirala A, Walsh KB, Wang Z, McCarthy C. Deep learning for real-time fruit detection and orchard fruit load estimation: Benchmarking of ‘MangoYOLO’. Precis Agric. 2019;20(6):1107–1135.

[31] Han TH, Kuo YF. Developing a system for three-dimensional quantification of root traits of rice seedlings. Comput Electron Agric. 2018;152:90–100.

[32] Yasrab R, Atkinson JA, Wells DM, French AP, Pridmore TP, Pound MP. RootNav 2.0: Deep learning for automatic navigation of complex plant root architectures. Gigascience. 2019;8(11):giz123. 31702012 10.1093/gigascience/giz123 PMC6839032

[33] Atanbori J, Montoya-P ME, Selvaraj MG, French AP, Pridmore TP. Convolutional neural net-based cassava storage root counting using real and synthetic images. Front Plant Sci. 2019;10:1516. 31850020 10.3389/fpls.2019.01516 PMC6888701

[34] Nair R, Strube M, Hertel M, Kolle O, Rolo V, Migliavacca M. High frequency root dynamics: Sampling and interpretation using replicated robotic minirhizotrons. J Exp Bot. 2023;74(3):769–786. 36273326 10.1093/jxb/erac427 PMC9899415

[35] Smith AG, Petersen J, Selvan R, Rasmussen CR. Segmentation of roots in soil with U-Net. Plant Methods. 2020;16(1):13. 32055251 10.1186/s13007-020-0563-0 PMC7007677

[36] Wang T, Rostamza M, Song Z, Wang L, McNickle G, Iyer-Pascuzzi AS, Qiu Z, Jin J. SegRoot: A high throughput segmentation method for root image analysis. Comput Electron Agric. 2019;162:845–854.

[37] Ronneberger O, Fischer P, Brox T. U-net: Convolutional networks for biomedical image segmentation. In: *Medical image computing and computer-assisted intervention – MICCAI 2015. MICCAI 2015. Lecture notes in computer science*. Cham: Springer; 2015. pp. 234–241.

[38] Seethepalli A, Dhakal K, Griffiths M, Guo H, Freschet GT, York LM. RhizoVision explorer: Open-source software for root image analysis and measurement standardization. AoB Plants. 2021;13(6):plab056. 34804466 10.1093/aobpla/plab056 PMC8598384

[39] Itzhaky Y, Farjon G, Khoroshevsky F, Shpigler A, Bar-Hillel A. Leaf counting: Multiple scale regression and detection using deep CNNs. Paper presented at: BMVC 2018. 29th British Machine Vision Conference; 2018 September; Newcastle, England.

[40] Lin T-Y, Goyal P, Girshick R, He K, Dollár P. Focal loss for dense object detection. Paper presented at: Proceedings of the 2017 IEEE International Conference on Computer Vision and Pattern Recognition (ICCV); 2018 October 22–29; Venice, Italy.

[41] Tan M, Pang R, Le QV. Efficientdet: Scalable and efficient object detection. Paper presented at: Proceedings of the 2020 IEEE/CVF Conference on Computer Vision and Pattern Recognition (CVPR); 2020 June; Seattle, WA, USA.

[42] Sindagi VA, Patel VM. Generating high-quality crowd density maps using contextual pyramid CNNs. Paper presented at: Proceedings of the 2017 IEEE International Conference on Computer Vision (ICCV); 2017 October 22–29; Venice, Italy.

[43] Kume T, Ohashi M, Makita N, Kho LK, Katayama A, Endo I, Matsumoto K, Ikeno H. Image analysis procedure for the optical scanning of fine-root dynamics: Errors depending on the observer and root-viewing window size. Tree Physiol. 2018;38(12):1927–1938. 30452737 10.1093/treephys/tpy124

[44] Paez-Garcia A, Motes CM, Scheible WR, Chen R, Blancaflor EB, Monteros MJ. Root traits and phenotyping strategies for plant improvement. Plan Theory. 2015;4(2):334–355. 10.3390/plants4020334 PMC4844329 27135332

[45] Yosinski J, Clune J, Bengio Y, Lipson H. How transferable are features in deep nNeural networks? Advances in neural information processing systems 27(NIPS ’14). Red Hook (NY): NIPS Foundation; 2014.

[46] Douarre C, Schielein R, Frindel C, Gerth S, Rousseau D. Transfer learning from synthetic data applied to soil–root segmentation in x-ray tomography images. J Imaging. 2018;4(5):65.

[47] Khoroshevsky F, Zhou K, Lazarovitch N. Dataset for “root length estimation: Automated minirhizotron image analysis with convolutional networks without segmentation” (version 1) [data set]. Geneva (Switzerland): CERN; 2022.

[48] Farjon G, Itzhaky Y, Khoroshevsky F, Bar-Hillel A. Leaf counting: Fusing network components for improved accuracy. Front Plant Sci. 2021;12: Article 575751. 34177972 10.3389/fpls.2021.575751 PMC8224400

[49] Khoroshevsky F, Khoroshevsky S, Markovich O, Granitz O, Bar-Hillel A. Phenotyping problems of parts-per-object count. In: European conference on computer vision. Cham: Springer; 2020. p. 261–278.

[50] Khoroshevsky F, Khoroshevsky S, Bar-Hillel A. Parts-per-object count in agricultural images: Solving phenotyping problems via a single deep neural network. Remote Sens. 2021;13(13):2496.

[51] Walter A, Silk WK, Schurr U. Environmental effects on spatial and temporal patterns of leaf and root growth. Annu Rev Plant Biol. 2009;60(1):279–304. 19575584 10.1146/annurev.arplant.59.032607.092819

[52] Machado R, Oliveira MDRG. Tomato root distribution, yield and fruit quality under different subsurface drip irrigation regimes and depths. Irrig Sci. 2005;24(1):15–24.

[53] Sharma SP, Leskovar DI, Volder A, Crosby KM, Ibrahim AMH. Root distribution patterns of reticulatus and inodorus melon (*Cucumis melo L*.) under subsurface deficit irrigation. Irrig Sci. 2018;36(6):301–317.

[54] Soda N, Ephrath JE, Dag A, Beiersdorf I, Presnov E, Yermiyahu U, Ben-Gal A. Root growth dynamics of olive (*Olea europaea L.)* affected by irrigation induced salinity. Plant Soil. 2017;411(1):305–318.

[55] Zhou K, Jerszurki D, Sadka A, Shlizerman L, Rachmilevitch S, Ephrath J. Effects of photoselective netting on root growth and development of young grafted orange trees under semi-arid climate. Sci Hortic. 2018;238:272–280.

[56] Guo LB, Halliday MJ, Siakimotu SJM, Gifford RM. Fine root production and litter input: Its effects on soil carbon. Plant Soil. 2005;272(1):1–10.

[57] Iversen CM, Childs J, Norby RJ, Ontl TA, Kolka RK, Brice DJ, McFarlane KJ, Hanson PJ. Fine-root growth in a forested bog is seasonally dynamic, but shallowly distributed in nutrient-poor peat. Plant Soil. 2018;424(1):123–143.

[58] Johnson MG, Tingey DT, Phillips DL, Storm MJ. Advancing fine root research with minirhizotrons. Environ Exp Bot. 2001;45(3):263–289. 11323033 10.1016/s0098-8472(01)00077-6

[59] Primka EJ IV, Adams TS, Buck AS, Eissenstat DM. Shifts in root dynamics along a hillslope in a mixed, mesic temperate forest. Plant Soil. 2022;477:707–723.

