# Supplementary material for: Automatic Root Length Estimation from Images Acquired In Situ without Segmentation
Source: Plant Phenomics. 2024 Jan 12;6:0132. doi: 10.34133/plantphenomics.0132 (PMC10790720; doi:10.34133/plantphenomics.0132)
Supplement: Supplementary 1 — Figs. S1 and S2 Tables S1 to S4 [file plantphenomics.0132.f1.zip › PlantPhenomics_D2300157f1.docx]

**Supplementary Materials**

**Fig. 1.** Annotated snapshot of the Rootfly graphical user interface (GUI). The image window located in the middle of the workspace displays the current image being interpreted and annotated. The window-pane located at the left of the workspace displays the various windows within the selected tube. The session pane located at the bottom of the workspace displays the various sessions and dates related to the experiment. The properties pane located on the right of the workspace displays the properties associated with the roots of a current session. The coordinate information of the points along the roots can be accessed by selecting the **"Debug**" subkey, and then selecting **"Display Root data"**. A dialog box will pop up and present the coordinate information of all roots in the selected observation tube.


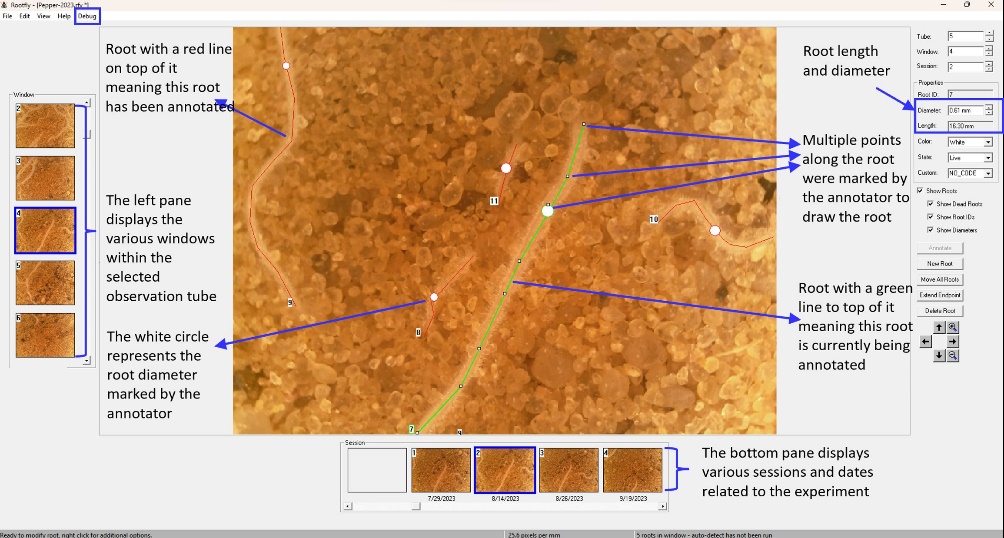


**Fig. 2.** Schema of the network modules [‎39]. (a) modules for generating a multiscale image representation with the “Backbone” module. (b) architecture of the direct regression module of the regression model—the subnetwork that is followed by the “Backbone”. (c) architecture of the modules that follow the “Backbone” in the points model.

(a)


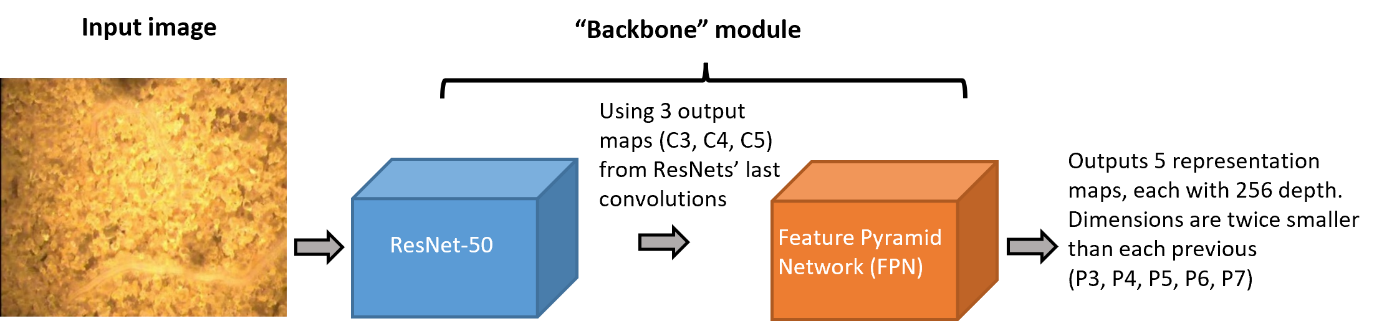


(b)


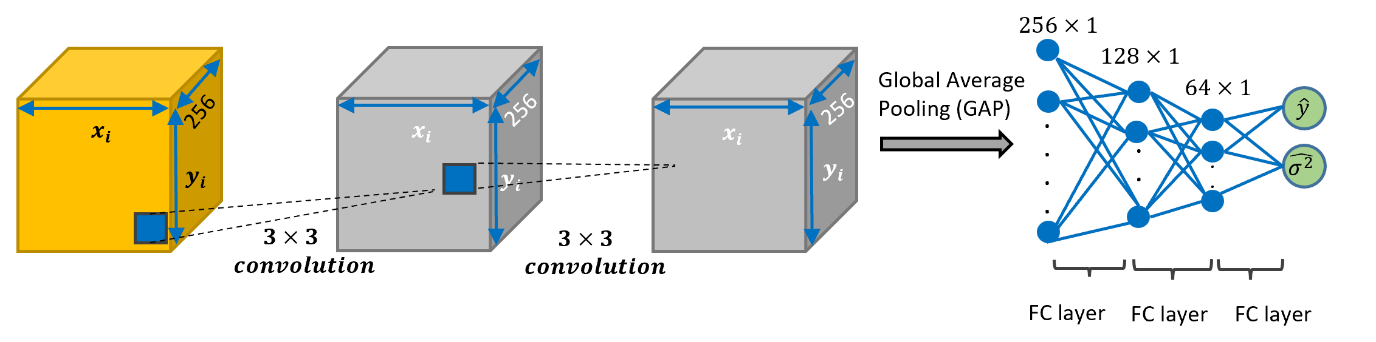


(c)


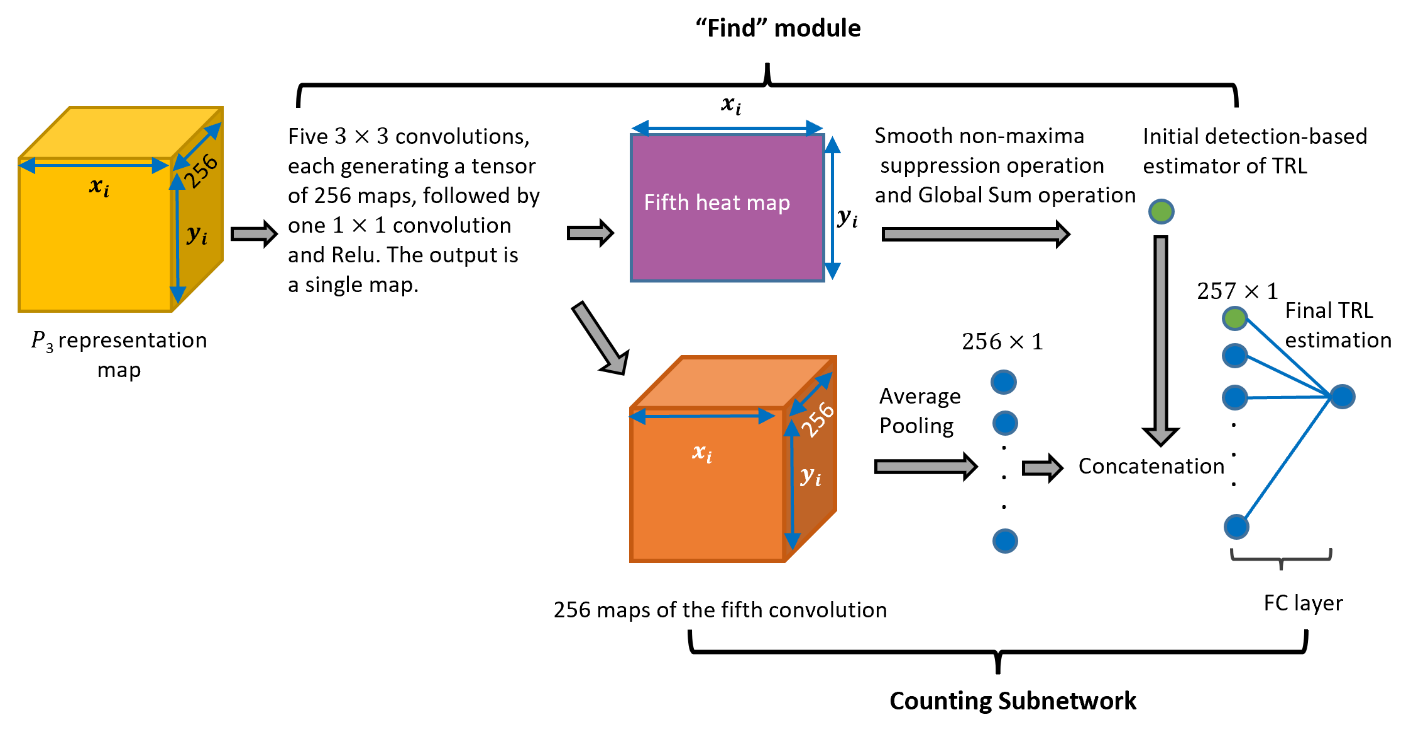


**Table 1.** A summary of the two suggested models' architectures. All details can be found in Itzhaky et al. [‎39].

| The Regression model | The points model |
| --- | --- |
| The "Backbone" in both models is a dense CNN used to generates five representations of the original input image, on multiple scales, designated P_3_–P_7_. | |
| - Each of the five image representations output by *“Backbone”*, *P_3_–P_7_*, are sent to a direct regression module so that multiple TRL estimates are produced based on different resolutions of the image representations. - This regression module contains two 3 × 3 convolutional layers, which output 256 maps with the same spatial dimensions as its input. - It is followed by a Global Average Pooling (GAP) layer, which flattens the maps to a 256 × 1 representation vector that is fed into two fully connected (FC) layers of decreasing size (128 and 64, respectively). - For each scale, following additional FC layer, this module yields two outputs: the expected TRL value ($\hat{y}$), and its variance ($\hat{\sigma^{2}}$). Among the five TRL estimates, the one with the lowest predicted variance for the input image is chosen as the final estimated TRL for that image. | - Following the *“Backbone”* module, there are the *“Find”* module followed by the “Counting subnetwork”. Only the high-resolution pyramid scale (*P_3_*) is used as input (and not all five $P_{i}$ tensors) to the *"Find"* module. Its output is a map at the same resolution, stating the probability of the presence of a point at each spatial location. - The *“Find”* module consists of four convolutional layers with 256 filters each, and outputs a single map by a fifth convolutional layer with one filter. - The internal layers are also guided by generating an intermediate heat map of points’ coordinates after each convolutional layer, with a decreasing kernel size. - The fifth heat map is subjected to smooth non-maximum suppression, keeping the activity of the values close to 1, while all other values in the map are reduced to 0. The coordinates of the non-zero values in the map are the predicted points’ coordinates of the roots. A Global Sum operation is then applied on top of this map, providing an additional feature to the following “Counting Subnetwork” module (instead of being an initial detection-based estimate as in the counting task). - The “Counting Subnetwork” follows the "Find" module. The input is the feature generated by the "Find" module based on the heat map and the feature tensor (with 256 depth) preceding it, from the “Find” module. A GAP operation is applied to the tensor, generating a 256 × 1 vector, which is concatenated with the initial detection-based estimate, resulting in a 257 × 1 features vector. The final TRL estimate is computed with FC layer from this vector. |

**Table 2.** Description of root image datasets, referring to the MR system type, crop, dataset size, and information on data partitioning to the training, validation, and testing sets.

| MR system | Dataset | Experiment name | Total number of images | Images without roots | Training images | Validation images | Test images |
| --- | --- | --- | --- | --- | --- | --- | --- |
| Manual | *Dataset 1* | Melon 2018 | 175 | 74 | 126 | 18 | 31 |
|  |  | Melon 2019 | 199 | 84 | 145 | 18 | 36 |
|  |  | Tomato 2019 | 200 | 82 | 146 | 18 | 36 |
|  |  | Tomato 2020 | 199 | 7 | 146 | 17 | 36 |
|  |  | Corn 2020 | 181 | 44 | 135 | 16 | 30 |
|  |  | Pepper 2021 | 189 | 53 | 138 | 17 | 34 |
|  |  | **Total for *Dataset 1*** | **1143** | **413** | **836**  **(318 without roots)** | **104**  **(36 without roots)** | **203**  **(59 without roots)** |
| Automated | *Dataset 2* | Pepper 2021 | 420 | 37 | **302**  (26 without roots) | **41**  (4 without roots) | **77**  (7 without roots) |
| Automated | *Dataset 3* | Pepper 2021 | 832 | 184 | - | - | - |
| Manual | *Dataset 4* | Melon 2018 | 521 | 300 | - | - | - |
|  |  | Melon 2019 | 217 | 77 | - | - | - |
|  |  | Tomato 2019 | 364 | 92 | - | - | - |
|  |  | Tomato 2020 | 178 | 91 | - | - | - |
|  |  | Corn 2020 | 116 | 31 | - | - | - |
|  |  | Pepper 2021 | 224 | 39 | - | - | - |

**Table 3.** Description of root image datasets—manual and automated cameras.

| Camera type | Dataset | Total number of images | Images without roots | Experiment name | Crop type | Stress type | Soil Type |
| --- | --- | --- | --- | --- | --- | --- | --- |
| Manual | *Dataset 1* | 175 | 74 | Melon 2018 | Melon | Low temperature | Sand |
|  |  | 199 | 84 | Melon 2019 | Melon | Low temperature | Sand |
|  |  | 200 | 82 | Tomato 2019 | Tomato | Salt stress | Sand |
|  |  | 199 | 7 | Tomato 2020 | Tomato | Salt stress and root restriction | Sand |
|  |  | 181 | 44 | Corn 2020 | Corn | Drought | Sand |
|  |  | 189 | 53 | Pepper 2021 | Pepper | Nitrogen deficiency | Loamy sand |
| Automated | *Dataset 2* | 420 | 37 | Pepper 2021 | Pepper | Nitrogen deficiency | Loamy sand |
| Automated | *Dataset 3* | 832 | 184 | Pepper 2021 | Pepper | Nitrogen deficiency | Loamy sand |
| Manual | *Dataset 4* | 217 | 77 | Melon 2019 | Melon | Low temperature | Sand |
|  |  | 116 | 31 | Corn 2020 | Corn | Drought | Sand |

**Table 4.** Results of testing the points model on the test set of Dataset 1 when it was initially trained on images from Dataset 2, but with additional training using randomly chosen images from Dataset 1. This was compared with the original results when the model was both trained and tested on the same data type (Dataset 1).

|  | $\vert\Delta RL\vert$ | MRD (GT>0) | $NRMSE$ | $R^{2}$ | $1-FVU$ |
| --- | --- | --- | --- | --- | --- |
| **Original results – training on *Dataset 1*** | **3.19** | **20.9%** | **0.041** | **0.958** | 0.95 |
| **Training on *Dataset 2*** | **7.45** | **39.0%** | **0.083** | **0.839** | 0.78 |
| Adding 10 images | 5.38 | 31.3% | 0.056 | 0.927 | 0.903 |
| Adding 20 images | 4.94 | 30.6% | 0.049 | 0.941 | 0.923 |
| Adding 40 images | 4.49 | 30.0% | 0.044 | 0.947 | 0.939 |
| Adding 60 images | 4.26 | 28.1% | 0.045 | 0.946 | 0.937 |
| Adding 80 images | 4.32 | 27.8% | 0.046 | 0.946 | 0.934 |
| Adding 100 images | 4.71 | 26.3% | 0.052 | 0.938 | 0.915 |
| Adding 120 images | 4.41 | 25.8% | 0.047 | 0.941 | 0.931 |
| Adding 140 images | 4.16 | 25.5% | 0.043 | 0.947 | 0.940 |
| Adding 160 images | 4.57 | 25.5% | 0.049 | 0.953 | 0.925 |
| Adding 180 images | 4.07 | 24.1% | 0.042 | 0.951 | 0.945 |
| Adding 200 images | 4.02 | 24.0% | 0.039 | 0.955 | 0.953 |
